# Modelling Mixed Microbial Culture Polyhydroxyalkanoate Accumulation Bioprocess towards Novel Methods for Polymer Production Using Dilute Volatile Fatty Acid Rich Feedstocks

**DOI:** 10.3390/bioengineering9030125

**Published:** 2022-03-21

**Authors:** Alan Werker, Laura Lorini, Marianna Villano, Francesco Valentino, Mauro Majone

**Affiliations:** 1 Promiko AB, Briggatan 16, 23442 Lomma, Sweden; 2 School of Chemical Engineering, The University of Queensland, Brisbane, QLD 4072, Australia; 3 Department of Chemistry, Sapienza University of Rome, Piazzale Aldo Moro 5, 00185 Rome, Italy; laura.lorini@uniroma1.it (L.L.); marianna.villano@uniroma1.it (M.V.); mauro.majone@uniroma1.it (M.M.); 4 Department of Environmental Sciences, Informatics and Statistics, Cà Foscari University of Venice, Via Torino 155, 30172 Venice, Italy; francesco.valentino@unive.it

**Keywords:** polyhydroxyalkanoates (PHA), polyhydroxybutyrate (PHB), mixed microbial cultures, activated sludge, respiration kinetics, Monod kinetics, oxygen mass balance, hysteresis, process modelling

## Abstract

Volatile fatty acid (VFA) rich streams from fermentation of organic residuals and wastewater are suitable feedstocks for mixed microbial culture (MMC) Polyhydroxyalkanoate (PHA) production. However, many such streams have low total VFA concentration (1–10 gCOD/L). PHA accumulation requires a flow-through bioprocess if the VFAs are not concentrated. A flow through bioprocess must balance goals of productivity (highest possible influent flow rates) with goals of substrate utilization efficiency (lowest possible effluent VFA concentration). Towards these goals, dynamics of upshift and downshift respiration kinetics for laboratory and pilot scale MMCs were evaluated. Monod kinetics described a hysteresis between the upshift and downshift responses. Substrate concentrations necessary to stimulate a given substrate uptake rate were significantly higher than the concentrations necessary to sustain the attained substrate uptake rate. A benefit of this hysteresis was explored in Monte Carlo based PHA accumulation bioprocess numerical simulations. Simulations illustrated for a potential to establish continuous flow-through PHA production bioprocesses even at a low (1 gCOD/L) influent total VFA concentration. Process biomass recirculation into an engineered higher substrate concentration mixing zone, due to the constant influent substrate flow, enabled to drive the process to maximal possible PHA production rates without sacrificing substrate utilization efficiency.

## 1. Introduction

Polyhydroxyalkanoates (PHAs) are a family of semi-crystalline polyesters that are naturally accumulated by many species of bacteria, for carbon and energy intermediate storage [1]. Accumulated polymer is stored in bacteria as intracellular granules. The recoverable pure polymers are biodegradable and have thermoplastic properties of interest for bioplastics and chemical industries [2]. PHA properties can be manipulated based on monomer composition, monomer distribution, and polymer molecular mass distribution. As such, PHAs are tagged as a biobased renewable resource with broad potential for services and products of value for society [3].

Limited types and amounts of PHAs are available commercially today [4], with supplies principally generated by pure culture production methods and refined feedstocks. PHAs can also be produced as a by-product of organic residual (waste) management using pure microbial cultures, as well as with open cultures of bacterial biomass that are enriched with the PHA-storing phenotype [5,6]. Open culture bioprocess environments can generate selection pressures. These selection pressures can favour preferred survival of populations of species of PHA-accumulating microorganisms due to competitive advantage with rapid substrate uptake and metabolism via PHA storage. Bioprocesses by design, or by coincidence, will generate selection pressures, for PHA-accumulating microorganisms, related to factors such as substrate type and feeding methods [7], operating temperature [8], and/or the process environments engineered for wastewater contaminant removal [9,10]. Significant storage capacities of PHAs in open or so-called mixed microbial cultures (MMCs) have been accomplished in the laboratory and up to pilot scale in the range from 40 to 90% gPHA per gram of biomass volatile solids [11,12]. The present work concerns bioprocess challenges for industrial scale PHA production using MMCs. Specifically, this work addresses the idea to develop bioprocess methods which allow producing PHA in MMCs with continuous influent flow constant volume bioprocesses using moderate to low concentration volatile fatty acid (VFA) rich feedstocks as substrate.

MMCs can be made to accumulate PHA with VFA rich feedstocks. Effluents of suspended solids separated acidogenic fermentation liquors from wastewater and sludges, dominated by (C2 to C5) VFAs, are typically found to result in co-polymer blends of poly(3-hydroxybutyrate-co-3-hydroxyvalerate) [13]. Other types of more complex MMC co-polymer blends have also been reported [14,15,16]. The VFA composition strongly influences the accumulated co-polymer monomer distribution and, thereby, resultant co-polymer blend crystallinity. Even full-scale waste municipal activated sludge has been shown to be directly applicable to produce commercial quality PHAs [17]. Evidence was given for engineering polymer properties and maintaining quality control with VFA feedstocks produced from fermented sludge and industrial waste at pilot scale. Extent of maximum polymer crystallinity was steered predictably over a wide range by the feedstock composition for consistently applied accumulation methods. Crystallinity influences microstructure with concomitant influence on thermal, physical, and mechanical properties to suit many kinds of applications.

Pilot scale projects have repeatedly demonstrated technical feasibility for commercial scale PHA production based on direct accumulation of surplus biomass treating wastewater or using biomass produced with selection pressures specifically for MMC PHA production [11]. Notwithstanding, specific considerations to address underlying expected challenges of bioprocess methods for industrial scale MMC PHA production are lacking in the research literature. Considerations of upscaled process have been given for acclimation towards augmented polymer storage [18], for mitigating influence of autotrophic flanking populations [19] and for influences of changes in process temperature [20] during the PHA accumulation, for examples. Robust industrial scale mixed culture PHA accumulation processes will be needed wherein methods are specifically independent, or at least insensitive, to feedstock VFA concentration. This independence would allow the same facility to have flexibility to produce polymers with “direct accumulation” [11] biomass fed with available regional VFA-rich feedstock. Such a facility would need to accommodate variability of feedstock VFA composition and concentration. Consideration of approach for industrial scale continuous flow through PHA accumulation, especially for cases with substrates of lower VFA concentrations (<10 gCOD/L), have not been found to be addressed in the research literature.

Pure culture methods for PHA production classically rely on fed-batch fermentation processes with concentrated feedstocks. A mass of organic substrate is to be delivered to a microbial biomass to achieve as high a titre of PHA containing bacteria as possible, in the shortest possible time, and within the limits of the available bioprocess volume [21]. Batch systems for pure culture PHA production methods may often be with starting broth organic substrate concentrations of between 10 and 20 g/L. The source feedstocks have an order of magnitude higher concentration. In this way, a high mass of substrate can be added with small additions of liquid volume. Ideas and methods for continuous production are evolving [22]. These continuous methods are applied to achieve gains in volumetric productivity if batch to batch down times are reduced. Higher concentrated feedstocks are nevertheless still desirable towards facilitating pure culture PHA process operations.

MMC PHA production methods must similarly strive to achieve as high a productivity as possible in mass polymer produced per unit volume and time. Acidogenic fermentation effluents can be sourced from commonly available regional sources of municipal and/or industrial organic wastewater and sludge. However, a challenge is that these typically available fermentation feedstocks do not often have high total VFA concentrations. VFA rich feedstocks from fermented organic waste streams from industrial and municipal sources may often, and in own direct experience with examples over 20 years, be in the low VFA concentration range between 0.5 to 10 g/L [23]. Higher VFA concentrations are possible with fermentations in selected limited cases such as dairy [24,25] and fishing [26] industry residues. However, these examples may not be sufficiently large enough VFA sources to promote wide generic implementation of MMC PHA production at industrial scale. For broad implementation of MMC PHA production, one must consider to exploit commonly available surplus organic residues that societies generate, collect and process. Bioprocess methods that are applicable over a wide range of VFA concentration are anticipated to offer greater flexibility to source a wider diversity of regionally available MMC feedstocks to produce PHA within the same generic industrial facility.

If VFAs are the product for transport and sale, then innovations in methods of VFA up concentration as part of recovery are required [27]. VFA extraction during acidogenic fermentation is furthermore reported to improve yields in acidogenic fermentation [28]. However, when PHA is the end product, MMC PHA accumulation processes are the up concentration unit process due to biochemical conversion (soluble VFA to PHA granules) and biomass separability. Therefore, so long as the dilute VFA rich feedstock supplies do not require undue transportation, it is only necessary that a sufficient VFA mass is available, even if it is in a dilute form. A sufficient VFA mass is to be delivered in a volumetric flow at industrial scale to exploit the PHA storing potential of a MMC biomass such as surplus municipal activated sludge [17]. Activated sludge thickened (concentrated) biomass solids, with at least 5 to 10% dry solids content, may be brought within reasonable distance to the site of PHA production where available VFA sources exist. In the present work, MMC biomass respiration has been investigated for development of industrial scale PHA production methods with dilute VFA-rich feedstocks.

With dilute VFA-rich feedstocks it is anticipated that the feed volumes will be larger than the available bioprocess working volume. The feed volume must flow through the process, and it is required that the biomass is retained in the process by, for example, membranes [29] or gravity settling [17]. The effluent from the accumulation process should have as low a VFA concentration as possible to limit waste of substrate and effluent management costs. However, substrate uptake rate depends on substrate concentration. Therefore, high productivity, with high volumetric rates of PHA production, supposes high sustained process substrate concentrations with reference to the modelled process using Monod kinetics [30]. Higher steady state concentrations of substrate in the effluent, for flow through accumulation processes, trade-off the goal of greater substrate utilization efficiency with the goal of greater volumetric productivity.

In previous research, it was observed that traditional Monod kinetics could not adequately describe the dynamics of response of a MMC biomass during fed-batch PHA accumulation [31]. Consequently, methods of process for industrial scale accumulation were anticipated based on replicated observations of an interpreted hysteresis in the biomass dynamics of respiration with pulse wise substrate additions. Industrial methods were introduced, and these included mixing zones with higher substrate concentration for stimulating high substrate uptake rates, while maintaining these higher rates in mixing zones of significantly lower substrate concentrations. However, these previous observations were not systematically evaluated for enrichment MMCs from different sources. They were also not brought into a frame of a predictive process model.

The objective in the present work was to systematically evaluate and confirm a model for stimulation and attenuation of substrate uptake rates during PHA accumulation. Specifically, it was of interest to assess if a common model framework could be applied to two distinctly different MMCs enriched for PHA accumulation. The goal was to refine insight for conditions and principles of bioprocess engineering to maintain both high substrate utilization efficiency and optimal volumetric productivity during continuous feed mixed culture PHA production. The approach is intended to be flexible and to work well in cases for PHA production with low VFA concentration feedstock at industrial scale.

Dynamics of respiration for laboratory and pilot scale MMC enrichment cultures were characterized by oxygen and chemical oxygen demand (COD) mass balances as a function of initial substrate concentration. Acetic acid and acetate mixtures were used as the model substrate. The dynamic response of the biomass was translated into a process model that was applicable to both cultures. This process model was then applied by means of Monte Carlo numerical simulations to experiment with ideas of a constant volume, flow through, PHA production bioprocess design. The numerical simulations expressed how stimulation and maintenance mixing zones could, in principle, be applied for achieving high rates of PHA production while still maintaining low effluent substrate concentrations.

## 2. Materials and Methods

### 2.1. Biomass from a Lab-Scale SBR

A PHA-storing mixed microbial culture was produced in a 1.0 L working volume aerobic sequencing batch reactor (SBR) at room temperature (20 to 25 °C). A mechanical stirrer provided complete mixing of the culture medium without any settling, and oxygen was delivered through air pumps connected to an array of ceramic diffusors. The SBR was inoculated with activated sludge from the “Roma Nord” (Rome, Italy) full-scale municipal wastewater treatment plant (WWTP). At the time of the present study, the enrichment culture was established under steady state conditions for 3 months (12 h cycle and organic loading rate (OLR) of 8.5 gCOD/L/d). The biomass was characterized with a PHA accumulation potential in the range from 0.63 to 0.70 gPHA/gVSS. Full details of the SBR operations and performances are reported elsewhere [32].

The SBR cycle program sequence with timer controlled peristaltic pumps was as follows: (a) feast substrate feeding (10 min; 0.42 L), (b) feast reaction phase (150 min), (c) surplus biomass (mixed liquor) withdrawal (3 min; 0.50 L), (d) famine substrate feeding (5 min; 0.08 L), (e) famine reaction phase (552 min). The 1-day sludge retention time (SRT) was equal to the hydraulic retention time (HRT) due to no applied settling phase. The feast substrate was an 85:15 mixture (COD basis) of acetic and propionic acids in a mineral medium at 10.12 gCOD/L. The substrate was trimmed to pH 6.0 with NaOH from a 3.0 M stock solution. The famine substrate comprised ammonium sulphate at 7.33 g/L, and with 80 mL addition per cycle, giving a nitrogen loading of 249 mgN/d. A net excess nitrogen loading was established per cycle given a C/N balance of 34 gCOD/gN. The mineral medium composition was (mg/L): K_2_HPO_4_ (334), KH_2_PO_4_ (259), MgSO_4_· 7H_2_O (100), CaCl_2_· 2H_2_O (50), thiourea (20), Na_2_EDTA (3), FeCl_3_· 6H_2_O (2), H_3_BO_3_ (0.3), CoCl_2_· 6H_2_O (0.2), ZnSO_4_· 7H_2_O (0.1), MnCl_2_· 4H_2_O (0.03), NaMoO_4_· 2H_2_O (0.03), NiCl_2_· 6H_2_O (0.02) and CuCl_2_· 2H_2_O (0.01). Stability of feast and famine selection conditions were routinely monitored from dissolved oxygen (DO) trends (WTW Cell Oxi 3310).

The biomass source for replicate respiration response experiments in the present work was the withdrawn surplus biomass (c) after feast reaction (b) as described above. The 500 mL withdrawn mixed liquor was directed to an aerated vessel with the added nitrogen rich famine substrate that the wasted biomass would otherwise have experienced for the remaining cycle segments (d and e). Famine conditions were maintained for at least 8 h before the biomass was then used for replicate respiration experiments. Experiments were conducted at room temperature after the applied famine period and these experiments were completed within 26 h of the biomass withdrawal from the SBR. The famine condition for the biomass was confirmed from measured nitrogen uptake during famine, and consistent performance in PHA content cycling (Fourier transform infrared spectroscopy (FTIR) measurements). Famine biomass in the present work refers to the biomass after famine conditions were applied for at least 8 h.

### 2.2. Biomass from a Pilot-Scale SBR

The pilot scale biomass was produced in an SBR (120 L working volume) operated with automatic control (Labview, National Instruments, Austin, TX, USA). The SBR was inoculated with activated sludge from Treviso full-scale WWTP (northeast Italy). The PHA-accumulating biomass exhibited typical accumulating potential between 0.40 to 0.46 gPHA/gVSS. Full details of the pilot SBR operations and performances are reported elsewhere [33].

The selection SBR was fed with filtrate coming from fermentation of a 33:67 (%*v/v*) mixture of the organic fraction of municipal solid waste (OFMSW) and excess waste activated sludge (WAS). Both organic input streams were produced and managed within the Treviso municipality. The fermentate used to produce the biomass was characterized with 75% soluble COD as VFA, and a VFA composition dominated by butyric and acetic acids followed by propionic and other organic acids. The SBR was operated continuously (6-h cycle and 1 d HRT and SRT) with no biomass settling phase. Aeration, with linear membrane blowers (Bibus EL-S-250), provided oxygen and mixing. The 6-h aerobic cycle was as follows: (a) surplus biomass (mixed liquor) withdrawal (0.5 min), (b) delay (10 min), (c) feed (0.5 min), and (d) reaction (349 min). Temperature (22–25 °C, maintained with immersion heater) and pH (8.0–8.5) trends were monitored, but not controlled. OLR with VFA was 3.3 ± 0.7 gCOD/L/d with variations due to VFA level and composition fluctuations from the feedstock fermentation process [33].

Two-liter grab samples from the withdrawn surplus biomass were sent chilled by overnight courier from the pilot plant in Treviso to the laboratory of the present work at La Sapienza in Rome. The received biomass was stored at 4 °C and used for experiments within 24 h after its arrival. Prior to experiments, the stored famine biomass was acclimated by warming to room temperature (23 °C) over a few hours with continuous aeration and mixing. The famine condition for the biomass was confirmed with assessment for negligible biomass PHA content (FTIR measurements).

### 2.3. Pulse-Stimulation Respiration Experiments

A stock of mixed liquor with the famine biomass was maintained aerated and well-mixed at constant temperature (24 ± 1 °C). For each pulse-stimulation experiment a grab sample of mixed liquor of defined dilution and volume (50 to 100 mL) was mixed to target a biomass suspended solids concentration measured as VSS (volatile suspended solids). Dilutions were made with the same media (laboratory scale MMC) or matrix (pilot scale MMC) of the mixed liquor, containing no or negligible biomass and no added organic substrate. pH was at the level established in the respective SBRs and not adjusted. For the pilot scale mixed liquor, biomass suspended solids were separated by gravity separation and decantation to generate dilution media with the same matrix. The source of dilution media was maintained similarly to the famine biomass source with constant aeration and at the same temperature. Respiration experiments were conducted with and without constant aeration, and initial substrate concentrations over a range from 5 to 500 mgCOD/L. Biomass concentrations were in the order of 1 and 2 gVSS/L for the laboratory and pilot biomass experiments, respectively.

For each experiment, the defined mixed liquor volume containing famine biomass was disposed directly to a vessel with magnetic coupled stirrer (600 rpm). This vessel was maintained with a pre-set level of constant aeration (0 to 1 mL/min air). Air flow from standard aquarium pumps was regulated through a Dwyer Series VF flowmeter. DO and pH trends were logged starting upon mixed liquor addition to the vessel and for a period of pre-aeration lasting at least 2-min. Then, a defined volume (pulse) of substrate (0.20 to 2 mL) from a concentrated stock solution was injected as an impulse to the defined mixed liquor volume. The time point of substrate injection was tagged to the log file. DO and pH trends were followed until dissolved oxygen concentration increased again asymptotically upon substrate consumption. Such a pulse-stimulation experiment lasted anywhere from a few minutes to the better part of 1 h. This duration was expected to be dependent on the biomass source, the mass of substrate added, and the concentration of the biomass. The added aliquots of substrate stock solutions were defined mixtures of acetic acid and/or sodium acetate generated from 2 M stock solutions of acetic acid and sodium acetate trihydrate (Sigma-Aldrich > 99%), respectively.

### 2.4. Analytical Methods

Suspended solids from selected famine mixed liquor samples (1.5 mL Eppendorf) having negligible soluble COD were separated by microcentrifugation (RCF of 12,000× *g* for 10 min). The supernatant was decanted to another Eppendorf tube by a single inversion, and residual hanging liquid was carefully wicked out of the tube with tissue. The pellet was either resuspended back to 1.5 mL with deionized water, or it was dried overnight at 105 °C. The suspended solids concentration was evaluated based on chemical oxygen demand (COD), for the resuspended pellet solution. The mixed liquor supernatant was stored at 4 °C pending ammonia analysis. COD measurements were with Spectroquant^®^ (Merck, Milano, Italy) 25–1500 and 300–3500 mg/L kits and procedure in combination with a Spectroquant^®^ NOV60A spectrophotometer (Merck, Milano, Italy). Calibration was based on an acetic acid stock solution dilution series. The COD of the famine biomass was converted to volatile suspended solids (VSS) based on the assumed biomass composition of C_5_H_7_O_2_N with 1.42 mgCOD/mgVSS. Ammonium ions were measured on supernatant samples spectrophotometrically at 420 nm by the direct Nessler method [34]. PHA content for the laboratory (feast and famine) and pilot (famine) biomass was evaluated qualitatively by FTIR on the dried pellet. FTIR measurements were performed on ground dried biomass pellets with an Alpha ATR-FTIR (Bruker, Lund, Sweden) with diamond crystal as previously reported [35].

Dissolved oxygen and pH-millivolt signals were measured continuously during experiments and logged at 0.1 Hz (HQ40d, LDO101, PHC201, Hach Lange, Solna, Sweden). The pH-millivolt signal was 4-point calibrated (pH 4, 7, 9 and 10). Probe dynamic response characteristic was based on measured instantaneous shift-up and shift-down changes: pH was logged for the trends in shifts between 0.01 molar phosphate buffer solutions. Phosphate buffer solutions were prepared with KH_2_PO_4_ and K_2_HPO_4_ at pH 5 and 9. In a similar way, DO probe dynamics were estimated from trends by moving the probe between mixed non-aerated and mixed aerated SBR media solutions. The media solutions were at low (DO ≈ 0 mg/L, mixed, non-aerated, with biomass suspended solids) and high (DO = 8.3 mg/L mixed, aerated, without biomass suspended solids) steady state DO levels. Shift up and down trends were evaluated in triplicate, and the first order response time constants were estimated for the pH and DO probes from least squares non-linear regression analysis of the first order exponential asymptotic trends (Prism, Version 9, Graphpad Software, San Diego, CA, USA). The probe first order time constants were found to be 0.35 ± 0.02 $s^{-1}$ (pH), and 0.14 ± 0.02 $s^{-1}$ (DO). The logged pH and DO signals were processed by a Savitsky-Golay [36] second order 7-point filter for signal smoothing and the first derivatives. The interpreted actual trends of DO and pH were the measured signal values, corrected for probe response dynamics based on the empirically determined first order probe response time constants [37].

### 2.5. Dissolved Oxygen and Chemical Oxygen Demand Mass Balance

The model for the data analysis was based on a mass balance for dissolved oxygen and chemical oxygen demand concentrations. It considered a defined impulse addition of substrate to a defined amount of active biomass (VSS), in a constant volume system with constant aeration. The initial biomass was a famine biomass meaning that the biomass existed with a low background level of endogenous aerobic respiration after being subjected to a long period without exogenous organic substrate supply. The mass balance assumed that the famine biomass would exhibit a feast response. A feast response means a sudden increase in aerobic respiration levels due to the added organic substrate. This increased level of aerobic respiration was assumed to be due to substrate uptake for PHA storage only (no active biomass growth). In particular, exogenous supplied acetic acid is converted into intracellular stored polyhydroxybutyrate granules (PHB) during the feast response. DO concentration changes in the control volume were linked to processes of aeration, endogenous aerobic respiration, aerobic respiration in substrate consumption, and aerobic respiration on stored PHA:

(1) $$\frac{dC_{o}}{dt}=Q_{oa}-\left(Q_{oe}+Q_{os}+Q_{op}\right)$$

where *C_o_* is the DO concentration (mgO_2_/L) as a function of time (*t* in minutes), *Q_os_* is the DO uptake rate (mgO_2_/L/min) due to substrate consumption, *Q_op_* is the DO uptake rate (mgO_2_/L/min) due to activity on stored polymer, *Q_oe_* is the biomass endogenous respiration rate (mgO_2_/L/min), and *Q_oa_* is the DO supply rate (mgO_2_/L/min) due to constant air flow with coarse bubble aeration.

The specific substrate uptake rate (*q_s_*, mgCOD/gVSS/min) was assumed to be coupled to oxygen uptake rate by a constant (average) yield (*Y_os_*) for oxygen consumed per amount of substrate consumed (mgO_2_/mgCOD):

(2) $$q_{s}=\frac{q_{os}}{Y_{os}}=\frac{Q_{os}}{Y_{os}\;\cdot \;X_{a}}$$

where *q_os_* is specific oxygen uptake rate (mgO_2_/gVSS/min), and *X_a_* is the initial (active) biomass concentration (gVSS/L). DO supply rate, governed by the conditions of volume, aeration, and mixing, was:

(3) $$Q_{oa}=k_{a}\left(C_{o}^{*}-C_{o}\right)$$

where *k_a_* is the estimated aeration oxygen mass transfer rate (1/min) with constant air flow and volume, and $C_{o}^{*}$ is the apparent maximum oxygen (saturation) concentration (mgO_2_/L). Aerobic respiration activity on stored polymer was assumed to ensue upon depletion of exogenous substrate. As substrate supply becomes exhausted, respiration shifts rapidly to famine and the subsequent consumption of stored polymer [30]. A switching function was used to describe this shift from aerobic respiration on exogenous substrate to the initial level of respiration activity on the amount of stored PHA as endogenous substrate:

(4) $$Q_{op}=X_{a}\;\cdot \;q_{op}{\left(\frac{k_{s}}{k_{s}+C_{s}},\frac{C_{o}}{k_{o}+C_{o}}\right)}_{\min}$$

where *k_s_* is the Monod apparent affinity constant on substrate concentration (mgCOD/L), *k_o_* is the Monod apparent affinity constant on oxygen concentration (mgO_2_/L), *C_s_* is the exogenous substrate concentration (mgCOD/L) as a function of time, $q_{op}$ is the initial specific aerobic respiration rate on stored polymer (mgO_2_/gVSS/min), and (*a*,*b*)_min_ is minimum of *a* and *b*. Thus, maximum aerobic respiration levels required that DO concentration was not limiting.

Aerobic respiration rate (DO consumption rate) on polymer was expected to be a function of the amount of polymer stored by the biomass [30]. Respiration on stored polymer results in a decrease of the stored polymer content. However, it was only the initial respiration rate on stored polymer that was estimated as the exogenous substrate supply became depleted. Therefore, any decrease in polymer content directly after substrate consumption was neglected and it is only the initial estimated level of respiration on the maximum amount of polymer stored directly after substrate depletion represented by *Q_op_*.

For the COD mass balance, amount of polymer produced was determined from the average yield on substrate given polymer storage and assuming no active growth following similar previous model developments [38]:

(5) $$X_{p}=\left(1-Y_{os}\right)\;\cdot \;\left(C_{si}-C_{s}\right)$$

where *X_p_* is the PHA concentration as function of time (mgCOD/L), and *C_si_* is the initial substrate concentration (mgCOD/L).

Specific substrate consumption rate was represented by Haldane-Monod kinetics given sufficient dissolved oxygen concentration:

(6) $$q_{s}=q_{s}^{e}\;\cdot \;{\left(\frac{C_{s}}{k_{s}+C_{s}+\frac{C_{s}^{2}}{k_{h}}},\frac{C_{o}}{k_{o}+C_{o}}\right)}_{min}$$

where *k_h_* is the apparent Haldane substrate inhibition factor (mgCOD/L), and $q_{s}^{e}$ is the extant maximum specific substrate uptake rate (mgCOD/gVSS/min). Directly after substrate addition, a transient phase was observed. An extant maximum specific rate was used because of this lag period before the biomass responded in its upshift to the full extent of respiration and substrate uptake rates due to *C_si_*. The biomass extant maximum substrate uptake rate is a function of the stimulating (upshift) substrate concentration (*C_si_*) and time. The transient phase could be described explicitly as a function of time [39]. However, in the model development it was found to be simpler to maintain a time implicit representation for all equations, including the upshift lag phase kinetics:

(7) $$q_{s}^{e}=q_{s}^{m}\;\cdot \;\left(1-f_{i}\frac{k_{i}}{k_{i}+\left(C_{si}-C_{s}\right)}\right)$$

where $q_{s}^{m}$ is the maximum specific substrate uptake rate (mgCOD/gVSS/min), *f_i_* represents the initial respiration level with respect to the maximum reached substrate uptake rate (0 < *f_i_* < 1), and *k_i_* is the respiration induction substrate concentration constant (mgCOD/L). The initial substrate uptake ($q_{s}^{i}$) is therefore:

(8) $$q_{s}^{i}=q_{s}^{m}\;\cdot \;\left(1-f_{i}\right)$$

*k_i_* relates to the amount of substrate consumed by the biomass to reach $q_{s}^{m}$ with respiration rate initially starting from $q_{s}^{i}$ at time, *t* = 0. The maximum expressed substrate uptake rate $q_{s}^{m}$ was interpreted to be dependent on the initial substrate concentration (*C_si_*):

(9) $$q_{s}^{m}=q_{s}^{r}+q_{s}^{u}\;\cdot \;\frac{C_{si}}{k_{u}+C_{si}}$$

where $q_{s}^{r}$ is an inherent biomass capacity for substrate uptake, $q_{s}^{u}$ is a capacity for increase in substrate uptake rate, and *k_u_* is an apparent half-saturation constant (mgCOD/L) for an upshift in substrate uptake rate given *C_si_* as the peak (initial) exogenous substrate concentration.

Specific uptake rates were estimated based on the measured stock biomass (gVSS/L) and substrate (gCOD/L) concentrations that were corrected for the known media dilutions that were used for each respiration experiment. Model exploration for assessing the mass balance with measured DO concentration trends was performed with Berkeley Madonna (Version 10, Berkeley, CA, USA). Data analysis for estimation of model parameters, by nonlinear least squares regression analyses, was performed with custom scripts written in MATLAB (Version 2020b, Mathworks, Kista, Sweden), or with model equations defined and fitted by non-linear regression analyses in Prism (Version 9, Graphpad Software, San Diego, CA, USA).

### 2.6. Monte Carlo Accumulation Process Simulation

**Figure 1:**
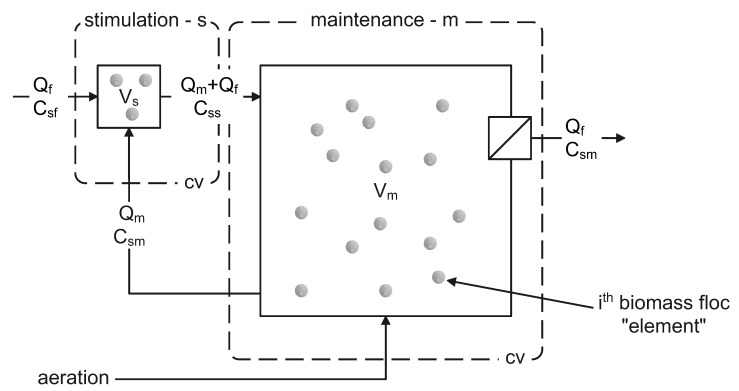
Model system for PHA accumulation comprising 2 process zones as completely mixed biomass stimulation and maintenance control volumes (cv), $V_{s}$ and $V_{m}$ respectively.

A Monte Carlo model was developed wherein a population of biomass suspended solid elements were modelled for PHA accumulation with no active microbial growth. Substrate concentration change as a function of time was calculated based on volumetric flow and mass balance differential equations for two connected well-mixed control volumes (Figure 1). This configuration represented a subsection of an idealised module for an industrial scale constant volume accumulation process. The process implements zones for stimulation and maintenance of biomass aerobic respiration levels as previously discussed [31]; biomass mixed liquor was continuously recirculated from a maintenance volume *V_m_* to a stimulation mixing zone *V_s_* receiving influent substrate. In *V_s_*, the influent substrate, and recirculated biomass mixed liquor, are mixed in proportions to reach elevated substrate concentrations to stimulate a maximal substrate uptake rate (Equation (9)). In *V_m_*, high substrate uptake rates are to be maintained at low substrate concentration (Equation (6)). The model represented a process subsection because in practice multiple parallel stimulation zones could be applied to service one larger, or several parallel, maintenance volumes in a larger volume industrial process.

In Figure 1, a feedstock with substrate concentration *C_sf_* is disposed to *V_s_* at a flow rate of *Q_f_* and the flow *Q_m_* maintains a defined continuous exchange and recirculation of the mixed liquor from the main volume *V_m_* to the mixing zone of *V_s_* and back again. This recirculation generates the concentration *C_ss_* in *V_s_*. The *V_m_* hydraulic residence time is *HRT_m_* = *V_m_/Q_m_*. Similarly, the maximum *HRT_s_* is *V_s_/Q_m_*.

Biomass suspended solids are retained in *V_m_* by an ideal separator. Process effluent from *V_m_* is at the influent flow rate of *Q_f_*. Effluent has substrate concentration *C_sm_*. Concentration *C_ss_* of substrate in *V_s_* is a function of *Q_f_*, *Q_m_*, *C_sm_* and *C_sf_* based on the flow and mass balances. Oxygen uptake rates were calculated and oxygen was assumed to be maintained at levels that were not limiting for microbial activity in *V_m_*. Influent flow rate was set to achieve a target organic loading rate with respect to the process theoretically maximum possible substrate uptake rate (Equation (9)).

The model applied the system of equations according to the above presented oxygen and chemical oxygen demand mass balances. Model parameters in Equations (1)–(9) used for the process simulations were determined as part of the biomass characterizations reported in the first part of the present investigation.

The respiration and substrate uptake rates for each individual biomass element was dependent on its history of exposure to dissolved substrate concentration. The capacity for substrate uptake rate for each biomass element was therefore determined by the highest most recently experienced substrate concentration (Equation (9)) in combination with the history of conditions of substrate concentration after that maximum substrate exposure (Equation (6)). The total process substrate uptake rate was determined by the sum of uptake rates for all the elements in the maintenance zone. Thus, substrate uptake rates in *V_s_* were assumed to be negligible due to anticipated DO limitation.

Uptake rates were driven with *k_u_* dependent up-shift kinetics (Equation (9)) for biomass elements seeing a history of constant or progressively increasing substrate concentrations. Substrate uptake rates were driven by *k_s_* dependent downshift kinetics (Equation (6)) for elements seeing a decrease in substrate concentration from a recent maximum value. For numerical stability, but also for practical representation based on previous work, the biomass elements were given a first order transient response time constant of 5 s. This meant that 95% of the expected response in respiration rate would be reached for a sustained change in substrate concentration after 15 s.

Numerical integration of governing differential equations was made for each time step to update stimulation “*s*” and maintenance “*m*” control volume substrate concentrations:

(10) $$\frac{dC_{ss}}{dt}=\frac{Q_{f}\;\cdot \;C_{sf}+Q_{m}\;\cdot \;C_{sm}-\left(Q_{m}+Q_{f}\right)\;\cdot \;C_{ss}}{V_{s}}$$

(11) $$\frac{dC_{sm}}{dt}=\frac{\left(Q_{m}+Q_{f}\right)\;\cdot \;C_{ss}-Q_{m}\;\cdot \;C_{sm}-Q_{f}\;\cdot \;C_{sm}-\sum_{i=1}^{n_{m}}x_{a}\;\cdot \;q_{si}}{V_{m}}$$

The element specific substrate uptake rates, $q_{si}$ (mgCOD/gVSS/min), were according to Equation (2) and with respect to the element mass $x_{a}$ (gVSS/element) for the elements found in $V_{m}$ at each time step. Haldane kinetics ($k_{h}$) and induction kinetics ($k_{i}$) were neglected due to the modelled process time scales and anticipated low substrate concentrations in *V_m_*.

The biomass elements were assumed to be ideally mixed in respective control volumes. Transport of biomass elements between $V_{m}$ and $V_{s}$ was made at each time step by random element selection and in proportion to flow rate and suspended solids concentrations. The biomass element statistics of residence time, respiration, polymer storage, and substrate uptake rate were estimated and evaluations of the process performance were made. The model and simulations were made with MATLAB (Version 2020b, Version 2020b, Mathworks, Kista, Sweden). Numerical integration of differential equations was with the ode45 solver, using a relative tolerance of $10^{-3}$, and a maximum time step of 0.05 s. The random number generator was uniquely seeded for each simulation run.

In keeping with previous work modelling MMC PHA production, the biomass maximum element specific substrate uptake rate $q_{si}^{m}$ (mgCOD/gVSS/min) was adjusted for PHA content [30]:

(12) $$q_{si}^{m}=q_{s}^{m}\;\cdot \;{\left(1-\frac{f_{PHAi}}{f_{PHA}^{m}}\right)}^{\alpha }$$

where $f_{PHAi}$ is the average PHA to active biomass mass ratio for the *i*th biomass element, and $f_{PHA}^{m}$ is the maximum possible PHA to active biomass ratio for the biomass. The inhibition exponent α is reported to range [30]. A value of 1.24 [40] was applied for these model simulations. The influent flow *Q_f_* was adjusted to provide a mass input rate of substrate based on *C_sf_* to give a constant substrate organic loading rate with respect to the maximum biomass uptake capacity (Equation (12)). Thus, the applied substrate organic loading rate was set to a relative constant level:

(13) $$Q_{f}\;\cdot \;C_{sf}=f_{L}\;\cdot \;\sum_{i=1}^{n_{m}}x_{a}\;\cdot \;q_{si}^{m}$$

where $f_{L}$ is the applied fraction of the maximum possible organic loading rate (0 < $f_{L}$< 1) with respect to the system maximum capacity in substrate uptake rate.

## 3. Results and Discussion

### 3.1. Laboratory and Pilot MMC Enrichment Biomass Characterization

Ten sets of experiments were performed with the laboratory scale SBR biomass harvested after the feast phase with a subsequent applied period of famine. All the respiration experiments on the laboratory scale famine biomass were spread over 3 months of steady state SBR operations. Two sets of replicate experiments were performed for the pilot scale MMC biomass harvested after famine. Consistency of the biomass condition before experiments was evaluated. FTIR measurements were performed on dried biomass samples from the laboratory biomass directly after feast harvesting. The laboratory and pilot famine biomass were also assessed by FTIR before experiments. Consistency of added ammonia uptake after laboratory feast biomass harvesting was confirmed.

**Figure 2:**
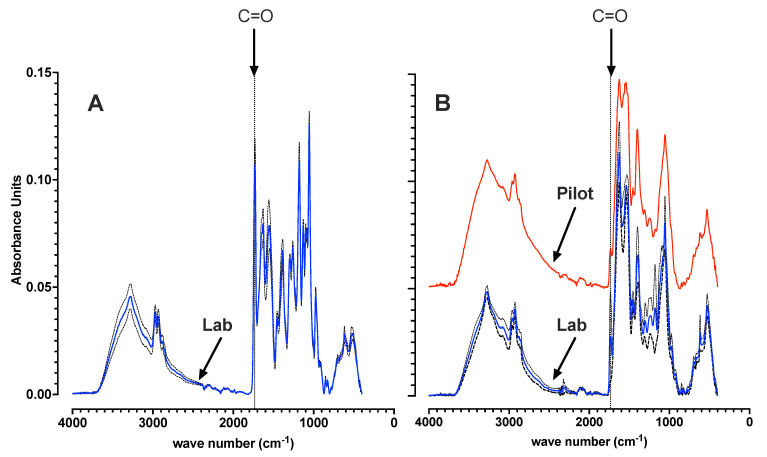
Background corrected and vector normalized FTIR spectra. Spectra are shown for the laboratory scale SBR dried biomass directly after biomass harvesting at the end of feast (**A**). Low PHA content was confirmed for laboratory and pilot famine biomass before start of respiration experiments (**B**). For the laboratory biomass, average spectra (solid line) are shown with standard deviation (dashed line) from biomass samples taken over the course of 10 distinct experiments carried out over 3 months. Significant or low PHA content is qualitatively represented by a characteristic large or small C=O absorbance peak (≈1735 cm${}^{-1}$). See [41] for further explanation.

Based on the experience from previously reported studies [35,41], FTIR measurements qualitatively confirmed a consistency of laboratory SBR operations with respect to post feast significant biomass PHA content, and a famine biomass with low to negligible PHA content (Figure 2). For the harvested laboratory biomass, progression to famine aerobic respiration by consumption of stored polymer was facilitated with added ammonia-nitrogen during 8 h of famine. Levels of ammonia after famine were 26.0 ± 3.5 mgNH_4_-N/L down from 62 mgNH_4_-N/L added just after feast harvesting. The pilot scale biomass was harvested after the famine cycle on site. Pilot scale famine biomass was similarly confirmed by FTIR to contain negligible PHA content before the start of the respiration experiments (Figure 2).

#### 3.1.1. Model Evaluation with No Active Aeration

**Figure 3:**
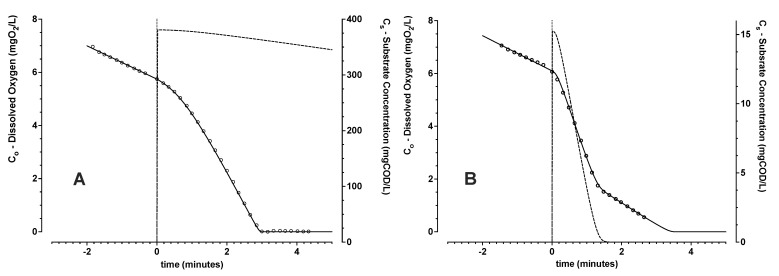
Trends of measured (∘) and modelled (solid line) dissolved oxygen concentration, as a function of time for *C_si_* levels starting (*t* = 0) at 381 (**A**) and 15 (**B**) mgCOD/L. Modelled trends of COD concentration *C_s_* (dashed line) from the mass balance are shown. These batch respiration experiments were conducted without active aeration using laboratory scale SBR enrichment biomass.

The oxygen and COD mass balance model was first evaluated with the laboratory scale SBR biomass in experiments with no active aeration (*k_a_* ≈ 0) (Figure 3). Respiration rates were always referenced to the initial observed endogenous respiration rate. Any minor contribution of passive oxygen transfer would introduce a slight negative bias to the estimation of the reference background level of endogenous respiration rate.

For initial substrate concentrations (*C_si_*) larger than about 20 mgCOD/L, dissolved oxygen levels were insufficient for complete removal of added substrate (Figure 3A). For these larger starting substrate COD levels, a progressively increasing respiration rate from an initial starting level was observed. This induction of increase in respiration rates above the reference endogenous levels, could be described implicitly in time by applying respective parameter constants of *f_i_* and *k_i_* given in Equations (7) and (8). Further, it was possible to establish that *k_o_* was negligible (<0.1 mgO_2_/L) for this MMC biomass dominated by finely dispersed suspended solids. A non-readily settling biomass was expected because enrichment was applied without requirement for settling as part of the selection pressure (HRT = SRT).

For lower starting *C_si_* levels (<20 mgCOD/L), initial dissolved oxygen concentrations were high enough to support complete added substrate COD removal (Figure 3B). Parameters describing the trends of respiration on the added substrate mass and kinetics for complete substrate removal could be readily estimated. Experiments replicated with 3 distinct batches of surplus biomass for *C_si_* concentrations of 5, 10 and 15 mgCOD/L gave an average oxygen yield on substrate *Y_os_* of 0.23 ± 0.03 (*n* = 7) gO_2_/gCOD. This estimated yield coincides with the previously reported theoretical *Y_os_* level of 0.26 gO_2_/gCOD [42] for PHA storage. Therefore, the resultant estimated yield of oxygen consumed for substrate removed supported the assumption of polymer storage without active biomass growth. This interpreted polymer storage dominating response was notwithstanding availability of ammonia-nitrogen and ortho-phosphate in the medium.

Transport of acetate into the cell and its activation is estimated to cost 1 ATP per Cmol of acetate [43]. Acetate transport into the cell can be both passive and active depending on substrate concentration and/or external conditions of pH [44]. Thus, conditions that promote for greater extent of passive transport would result in reduced oxygen demand for PHA accumulation, and consequently *Y_os_* values can also be lower than the estimated theoretical level of 0.26 gO_2_/gCOD.

A consistently low *k_s_* value of 0.110 ± 0.004 mgCOD/L was estimated for the cases with low starting substrate concentrations. A low apparent substrate affinity constant (*k_s_*) was expected. In previous work [40], an arbitrarily low *k_s_* value of 0.2 Cmmol/L (6.2 mgCOD/L) was selected, and not estimated explicitly to avoid numerical problems in other parameter determination. The present work suggests an affinity constant for acetate in the substrate consumption (Equation (6)) that is an order of magnitude lower.

#### 3.1.2. Model Evaluation with Active Aeration

**Figure 4:**
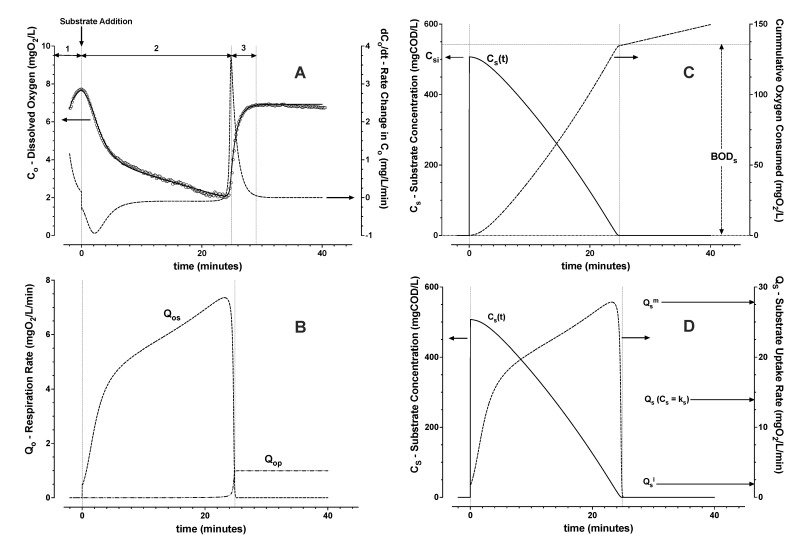
Typical example for trends of measured (∘) and modelled (—) dissolved oxygen (**A**) with Zone 1 (pre-aeration), Zone 2 (substrate consumption), and Zone 3 (re-aeration). From estimated oxygen mass transfer rate (*k_a_*, Zone 3), and endogenous respiration *Q_oe_* (Zone 1), respiration rates on substrate and polymer were calculated (**B**). Average yield *Y_os_* was given by estimated oxygen demand (*BOD_s_*) for added substrate removal (**C**). Trends of substrate uptake rate could thereby be estimated from the mass balance (**D**).

Assessment of biomass respiration response over a wider range of initial substrate concentration required oxygen supply. Constant aeration maintained dissolved oxygen levels greater than 1 mgO_2_/L, and, thereby, they were significantly greater than the estimated *k_o_*. The model represented observed trends of dissolved oxygen, for an initial substrate concentration (Figure 4). Even if substrate concentration as a function of time was not measured explicitly, the endpoint of substrate consumption was implicitly determined according to the model by the maximum in the rate of change (inflection point) of dissolved oxygen concentration given constant air flow rate. When substrate becomes exhausted, oxygen levels will increase first rapidly and then asymptotically to a higher dissolved oxygen level due to the shift to lower respiration rates.

**Figure 5:**
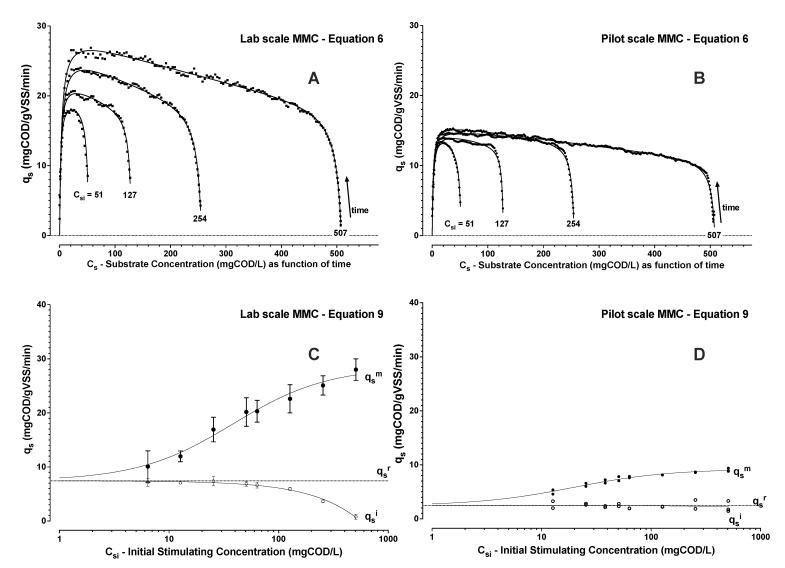
Figures show typical results for the laboratory (**A**) and pilot (**B**) scale MMCs experiments with respective trends of interpreted specific substrate uptake rate (*q_s_*) as a function of *C_si_*, and with substrate concentration changing implicitly in time. Points (•) correspond to the rates and concentrations derived from DO concentration data and the trend lines are the curves fit by least squares regression analysis to Equation (6). Results of estimated initial (∘, $q_{s}^{i}$) and maximum (•, *$q_{s}^{m}$)* specific substrate uptake rates for laboratory (**C**) and pilot (**D**) scale MMCs are shown as a function of initial acetic acid substrate concentrations (*C_si_*). The trend of maximum substrate uptake rate follows Equation (9) by least squares regression analysis. The initial specific substrate uptake rate decreased linearly with increasing initial acetic acid concentration.

Each experimental data set was divided into 3 zones for the model parameter estimation (see Figure 4A): Zone 1—pre-aeration before substrate addition, Zone 2—substrate consumption after its sudden pulse wise addition, and Zone 3—post aeration after the above mentioned inflection point. Only the first 4 min of Zone 3 were included in the data analysis. In this way, the re-aeration trend with the average (approximately constant) respiration rate on the maximum level of stored polymer was estimated. To avoid a risk for uncertainty in parameter value prediction, a progressive approach towards robust step-wise parameter estimation was applied. The 8 parameters (*k_a_*, *f_i_*, *k_i_*, *k_s_*, *k_h_*, *Y_os_*, *Q_op_*, $q_{s}^{m}$) were estimated in 6 steps:

1. From Zone 3, the re-aeration *C_o_* trend (Equations (1) and (3)) was fit by non-linear least squares regression with assumption of constant *Q_oe_* and *Q_op_*. From the fitted trend, the oxygen mass transfer coefficient *k_a_* was estimated;
2. From Zone 1, the average reference level of endogenous respiration, *Q_oe_* (Equations (1) and (3)), was then estimated assuming negligible *Q_op_*;
3. From Zone 3, *Q_op_*, the average respiration rate on stored polymer directly after substrate depletion, was then calculated (Equations (1) and (3));
4. From Zone 2, the trend of respiration rate on substrate, *Q_os_*, was then estimated (Equations (1) and (3));
5. From Zone 2, the integral of *Q_os_*, the cumulative biochemical oxygen demand (*BOD_s_*) due to substrate removal, was estimated. Then the average yield *Y_os_* and the trend of substrate concentration were calculated (Equation (2));
6. From Zone 2, the trend of substrate uptake rate as a function of estimated substrate concentration was then used to determine remaining parameters (Equations (6)–(8)). The induction and downshift substrate affinity constants (*k_i_* and *k_s_*, respectively) were interpolated from the derived trend of substrate uptake rate as a function of interpreted substrate concentration. Remaining parameters were estimated by nonlinear least squares regression analysis (see Figure 4 and Figure 5).

The laboratory scale SBR biomass was characterised in triplicate with distinct famine biomass batches over a week of steady SBR operations (Figure 5A,C). The pilot scale SBR biomass was similarly characterized with replicate evaluations from one grab sample of the pilot surplus activated sludge (Figure 5B,D). Summary of experiments and outcomes for the biomass evaluations with respect to the parameters of the model (Equations (1)–(9)) are given in Table 1 and depicted in Figure 5.

The estimated average yield of oxygen on substrate *Y_os_* (Table 1) reproduced outcomes reported above for cases without active aeration. *Y_os_* for the pilot biomass was also found to be close to theoretical predictions for microbial activity due to polymer storage only [42]. The assumption to neglect active growth in the model evaluation was therefore supported also for the pilot scale biomass. The oxygen mass transfer coefficient (*k_a_*) was determined independently for each experiment with consistent outcomes. A lower air flow rate was applied for the pilot scale biomass experiments due to lower levels of respiration rate.

A reproducible low substrate affinity constant (*k_s_*) was observed for both biomass sources. The *k_s_* value, about 2 mgCOD/L, was higher than the value estimated for experiments reported above without active aeration at low initial *C_si_* values. The *k_s_* (without active aeration) was reproducible and estimated by non-linear least squares regression model analysis. The *k_s_* (with active aeration) was estimated pragmatically as the interpolated concentration at half the maximum substrate uptake rate from the 6-step derived trend of *C_s_* with time. The value of about 2 mgCOD/L was considered to be a conservative estimate of what may actually be a lower affinity constant in reality. A *k_s_* of 2 mgCOD/L is still much lower than previously assumed [40]. *k_s_* has significant bearing on the performance of a flow through PHA accumulation bioprocess. A lower *k_s_* means, with all other parameters being equal, that similarly high polymer production rates can be maintained with lower substrate concentrations. Lower substrate concentration means less substrate lost in the effluent of a flow-through process giving a better substrate utilisation efficiency.

As expected, the maximum observed substrate uptake rates were dependent on the initial applied substrate concentration. Contrary to trends of substrate consumption, the affinity constant for stimulating an upshift to a maximum substrate uptake rate (*k_u_*) was found to be significantly larger than *k_s_* (Table 1). Thus, the hysteresis that was anticipated from previous work [31] was confirmed from the model that could be similarly applied for two distinct MMC cultures. Levels of substrate concentration that are necessary to stimulate to a maximum rate of substrate uptake are predicted to be higher than the concentration levels that are necessary to maintain a substrate uptake rate once it has been established. The upshift kinetics (Equation (9)) and downshift kinetics (Equation (6)) both similarly follow a Monod model but they did not follow the same path.

Enrichment MMC biomass sourced from laboratory and pilot SBRs revealed a similar behaviour, but the pilot MMC exhibited about one third the specific maximum rate to assimilate substrate. The trends of maximum substrate uptake rate further suggested an inherent starting (resting) capacity for substrate uptake rate ($q_{s}^{r}$). This capacity can be seen in the trends shown in Figure 5C,D. The maximum substrate uptake rates approached the initial inherent rates with decreasing *C_si_*. However, when dosing with acetic acid, Figure 5 also shows how the initial levels of substrate uptake rate ($q_{s}^{i}$) decreased below the estimated $q_{s}^{r}$ in direct proportion to added amounts of acetic acid.

**Figure 6:**
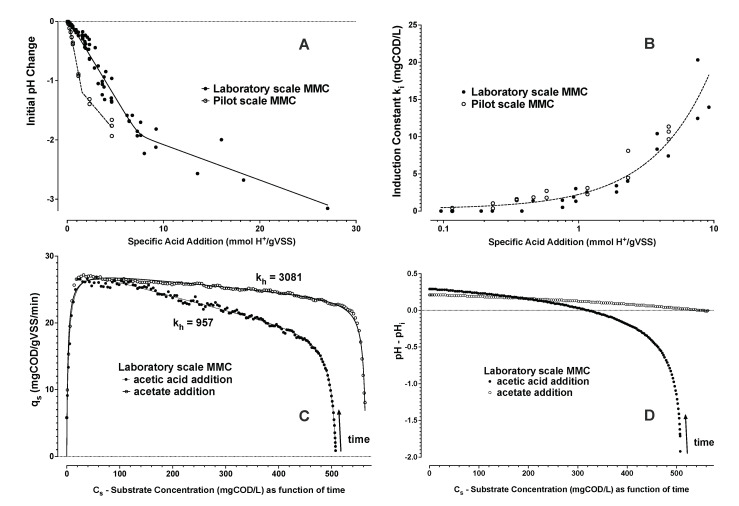
Initial pH changes are shown due to specific acid addition with the substrate (**A**). Corresponding to this acetic acid and/or acetate addition, the linear trend of influence (shown on a log scale) for the induction (lag phase) constant (*k_i_*) is similarly shown as a function of the specific acid addition (**B**). An example for the dynamics of substrate uptake rate during COD consumption as an implicit function of time for addition of acetic acid or acetate is given (**C**) with information of the measured trend in pH (**D**) with respect to the initial pH level (pH_i_) during the same time.

Higher initial levels of acetic acid suggested apparent substrate inhibition during substrate uptake, as depicted in Figure 5A,B and this inhibition could be described by applying a Haldane inhibition constant *k_h_* (Equation (6)). Since, the acetic acid addition included both organic substrate and acid addition, it was unclear if COD levels, and/or acid addition were causative factors to observed substrate inhibition. Additional respiration experiments were performed with the laboratory SBR biomass using acetic acid and acetate blends. These experiments suggested that, in the range of conditions tested, it was the amount of acid addition and not the initial COD concentration that promoted the decrease in $q_{s}^{i}$ with respect to $q_{s}^{r}$ (Figure 6B,C).

The buffer capacity of the mixed liquor involves contribution from dissolved solids as well as the biomass suspended solids. Initial pH change due to acid addition could be characterized with respect to the specific acid addition. The buffer capacity for mmol/L $H^{+}$ added with respect to *X_a_* concentration is shown in Figure 6A. Results suggested that even if the pilot SBR biomass exhibited lower buffer capacity (higher pH change for the same specific acid addition), similar levels of specific acid addition resulted in a similar induction lag phase described by *k_i_* (Figure 6B). Thus, the amount of specific acid addition causing sudden pH change may be of more direct influence on the initial biomass response to organic acid dosing rather than the absolute initial pH change. An influence of the amount of acid addition on uptake rate was also inferred by comparison of pH and uptake rate trends (Figure 6C,D). A lower *k_h_* (higher apparent Haldane substrate inhibition) was observed for acetic acid versus acetate additions in experiments for approximately the same starting COD concentration.

**Figure 7:**
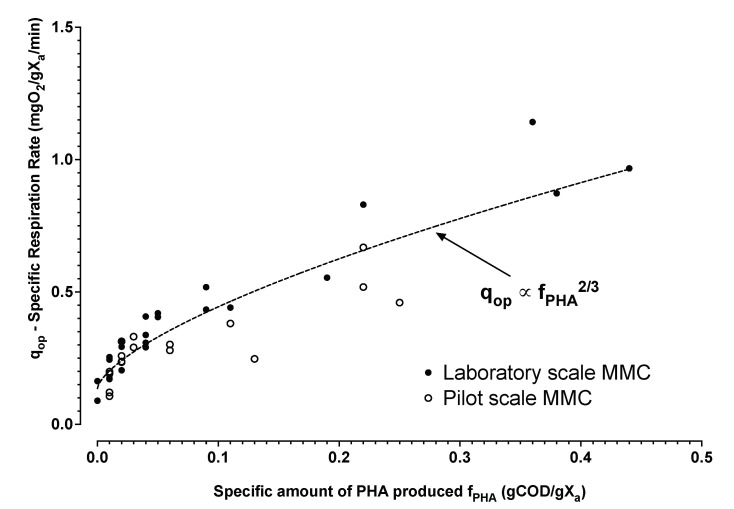
Estimated levels of biomass specific respiration rate after substrate consumption with respect to the estimated levels of PHA calculated from added substrate and yield of oxygen on substrate assuming no active biomass growth. The trend line is the least squares regression estimate for combined laboratory (•) and pilot (∘) data.

The relatively small increment in PHA content that accumulated in the biomass after a small pulse of substrate was not measured directly as part of the study. The median applied substrate pulse for the experiments was 57 mgCOD/L acetic acid concentration. From Table 1, and the COD mass balance (Equation (5)), the expected increment of biomass polyhydroxybutyrate content can be estimated. Assuming an active biomass concentration (*X_a_*) of 1 gVSS/L, 57 mgCOD/L of acetic acid would result in about 2.5 percent gPHA/gVSS. Even though the biomass PHA content was not measured explicitly, it was of interest to evaluate if the independently determined level of biomass respiration after substrate consumption corresponded to the model predicted level of PHA produced (*X_p_*). Directly after substrate consumption the residual elevated respiration rate *Q_op_* was estimated from Equations (1) and (3). *Q_op_* should be directly related to aerobic metabolism on stored PHA [30,38]. The residual respiration rate levels were found to follow an expected two-thirds power law trend (Figure 7) as a function of the estimated specific polymer concentration (*X_p_*/*X_a_*). This outcome suggests an internal model consistency and supports previous research findings for microbial activity during famine [30].

#### 3.1.3. Monte Carlo Simulation of PHA Accumulation in MMC Enrichment Biomass

Sets of PHA accumulation numerical simulations were performed to explore the implication of the interpreted stimulation-maintenance hysteresis due to upshift and downshift substrate affinities given by:

1. the stimulation of respiration rates due to the highest most recent substrate concentration as influenced by *k_u_* in Equation (9), and
2. the maintenance of an attained respiration rate with decreasing substrate concentrations as influenced by *k_s_* in Equation (6).

Simulations comprised an aerated maintenance volume *V_m_* of 500 L, containing 5000 mgVSS/L active biomass with PHA accumulation potential of 0.6 gPHA/gVSS. The biomass suspended solids were divided into $10^{5}$ individually followed floc elements ($x_{a}$ = 25 mgVSS active biomass per element). The estimated average kinetic parameters of the laboratory scale biomass were applied (Table 1). An influent feedstock with a low *C_sf_* of 1000 mgCOD/L was supplied continuously with adjusted flow *Q_f_* to maintain a targeted fraction of the biomass maximum substrate uptake rate ($f_{L}$). *Q_m_* flow rates were set to a defined *HRT_m_*, and *V_s_* was adjusted to ensure an *HRT_s_* of 30 s based on *Q_m_* in all cases. Simulations were maintained for up to 1 h modelled time by which time steady trends were well-developed and polymer content approached levels in the order of 0.3 gPHA/gVSS.

**Figure 8:**
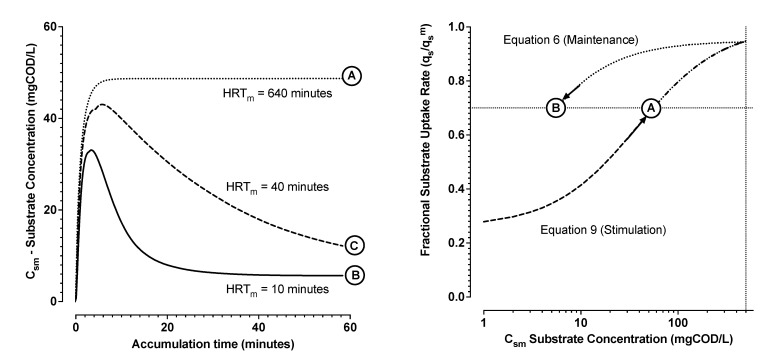
Simulation results for a fractional substrate loading rate of $f_{L}$ equal to 0.7 while varying the recirculation flow rate for higher and lower *HRT_m_* levels. Higher *HRT_m_* levels approach conditions of effective substrate feed directly into *V_m_* (A). For lower *HRT_m_*, influence of *C_ss_* uptake rate stimulation in *V_s_* enables for higher uptake rates maintained at lower *C_sm_* concentration (B). Moderate *HRT_m_* levels require longer times for all the biomass to be exposed to *C_ss_* in *V_s_* (C).

An influence of stimulation-maintenance hysteresis is illustrated in Figure 8 for cases of *HRT_m_* equal to 10, 40, and 640 min and a constant relative substrate loading rate of $f_{L}$ equal to 0.7 in all three cases. For increasing *HRT_m_*, the frequency that any given floc element recirculates to the *V_s_* influent mixing zone, decreases progressively. At longer *HRT_m_* the system approaches limiting conditions of *Q_f_* effectively flowing directly into *V_m_*. For influent flowing directly to *V_m_*, biomass respiration rates will initially increase due to increase of *C_sm_* in *V_m_* and the active biomass will respond with a corresponding increase in respiration rate (Equation (9)). *C_sm_* will increase and approach a steady state value when the substrate mass inflow rate matches the sum of the effluent mass outflow rate plus the biological mass uptake rate for PHA storage. Loading the modelled laboratory biomass at $f_{L}$ equal to 0.7 resulted in such a predicted steady state effluent substrate concentration of about 50 mgCOD/L with longer *HRT_m_*. In these cases, the process dynamics are driven only by Equation (9). The system settles, in this example, at “A” in Figure 8.

For reduced *HRT_m_*, flocs of biomass are more frequently and repeatedly stimulated to a higher respiration rate due to exposure to *C_ss_* in *V_s_*. Over a period of about 3 × *HRT_m_*, essentially all the biomass suspended solids should have been disposed to respiration stimulation in *V_s_*. Increasing *Q_m_* to give a 10-min *HRT_m_*, $f_{L}$ equal to 0.7, resulted in *C_ss_* in the order of 500 mgCOD/L in *V_s_*. This level is significant for stimulating maximal respiration rates for the laboratory scale biomass (Table 1). Initially, *C_sm_* substrate concentrations increased in *V_m_*, and two populations of biomass activity developed in parallel. Some flocs were respiring at a level due exposure to the increasing *C_sm_* levels in *V_m_* (Equation (9)). However, a second population of flocs developed increasingly over 3 × *HRT_m_*. These flocs had passed through *V_s_*. This population respired at higher levels due to contact to *C_ss_* in *V_s_* (Equation (9)), and subsequent *C_sm_* in *V_m_* (Equation (6)). In this way, the system settled at an order of magnitude lower steady state *C_sm_* in *V_m_* of 6 mgCOD/L (“B” in Figure 8). This lower effluent concentration was approached as all biomass elements passed through *V_s_* at least once. A similar overall level of substrate uptake rate resulted at “B” as for “A”, but with a significantly improved substrate utilization efficiency. By floc element exposure to respiration stimulation to near maximal rates in *V_s_* a higher respiration was maintained down to lower substrate concentrations (Figure 5A,C). For increasing *HRT_m_*, it takes longer for the system to reach steady state due a longer *V_m_* volumetric turnover time (“C” in Figure 8). Thus, lower *HRT_m_* served to stimulate all process biomass as quickly as possible.

#### 3.1.4. Future Research Perspectives and Challenges

This strategy for engineering accumulation bioprocess with internal, or external, recirculation and contact to elevated substrate concentrations that exist in the influent mixing zone(s) becomes increasingly more relevant the lower the influent substrate concentration becomes. Stimulation of respective biomass elements is asynchronous in time because not all biomass elements experience “stimulation” at the same time. Monte Carlo modelling can be applied as a way to understand the nature of the distribution of activities for discrete floc elements. In contrast, the typical approach for PHA accumulation with laboratory and pilot scale systems in the reviewed research literature [11,12] has been by dosing often concentrated substrate directly into a well-mixed aerated vessel containing PHA accumulating biomass. This is a synchronous strategy for stimulating high respiration rates because all the process biomass is subject to the same substrate concentration history at the same time.

Typically, dissolved oxygen [25,45,46], redox conditions [47], or pH [48] can be used with feedback control for timing the frequency of pulse feeding events. When substrate is concentrated, the process may be fed-batch and initiated with lower starting volume with a more concentrated biomass [49]. Furthermore, when the feedstock is concentrated then small pulse wise volume inputs can be added to bring the entire working volume quickly to substrate levels that result in maximum substrate uptake rates. Even in such cases, the process volume may not be sufficiently large to be able to supply enough VFA mass to reach saturation of PHA content in the process biomass. Intermittent settling and decanting cycles have been suggested as one strategy to extend the process capacity to enable delivery of more substrate [50]. However, periodic batch decanting of excess volume introduces a dead time to the accumulation process. This added time for intermittent settling and decanting is a disadvantage because it reduces volumetric productivity. Continuous feed MMC PHA production methods therefore need further attention for the industrial developments.

Additions of substrate in MMC accumulation cannot be too large due to a risk for inhibition [46]. Larger additions of substrate also increase the availability of substrate. Availability of substrate (without other limiting factors) can stimulate for active growth of the accumulating as well as flanking heterotrophic biomass populations [51]. Methods for dosing substrate based on pH control results in sustained elevated substrate concentrations [52] that are anticipated to contribute to stimulate for unwanted flanking growth. Continuous feeding methods for MMC production methods require to be developed and undertaken in ways that do not unduly reduce the yield of product formation by promoting unwanted flanking biomass growth.

Smaller pulse-wise substrate additions can be made in a fed-batch process with peak substrate concentrations that are just sufficient to stimulate high average substrate uptake rates [17]. This semi-continuous feeding approach can be performed with fed-batch or flow through process methods. However, fed-batch methods with many smaller pulses also introduce a dwell time between successive inputs. The dwell time between onset of substrate depletion from one pulse, and the control system triggering for the next pulse of substrate, has been observed in own developments to introduce a significant accumulated dead time with lower substrate uptake rate. This dead time decreases process volumetric productivity. More frequent interruption of substrate addition leading to punctuation of loss in respiration rate has been observed to furthermore risk to result in PHA of lower average molecular weight [31]. Therefore, the most optimal approach for MMC PHA accumulation is a process with continuous feeding that maintains the highest possible substrate uptake rate with the lowest possible effluent substrate concentration.

Methods supporting the development of industrial MMC PHA accumulation with continuous feed supply and as a flow through bioprocess have not been found to be reported in the research literature. Initial outcomes in published work for fed-batch methods applying constant feed based on predetermined rates without feedback control were not found to be satisfactory [46]. Since then pulse-wise feeding methods have become the standard practice in approach for MMC PHA production. Notwithstanding, continuous flow methods are desirable for continued advancements because they offer an optimal sustained volumetric productivity.

**Figure 9:**
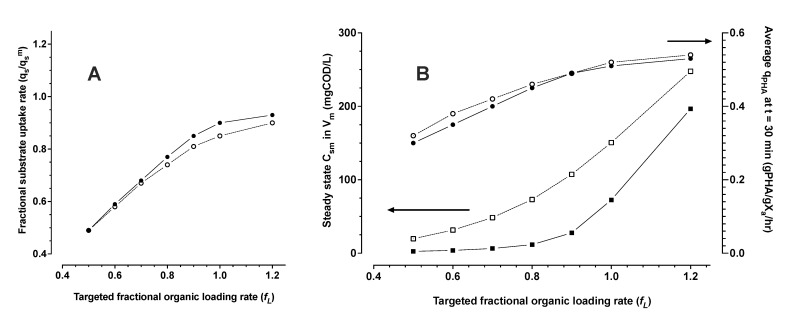
The targeted fraction of the system maximum possible substrate uptake rate (*f_L_*—see Equation (13)), versus the actual fraction of maximum uptake rate achieved in the process (**A**). Maintenance volume (*V_m_*) steady state substrate concentration *C_sm_* (squares), and average specific PHA production rates after 30 min accumulation (circles) as a function of the targeted fraction of the system maximum possible substrate uptake rate (**B**). Closed and open symbols are for *HRT_m_* of 5 and 250 min, respectively.

As an example, numerical simulations were performed to explore how the application of a stimulation zone could influence substrate utilisation efficiency while pushing the system towards theoretical limits of maximum PHA production rates with a feedstock VFA concentration of 1000 mgCOD/L (Figure 9). Higher applied organic loading rates drive the system biomass to maximum possible substrate uptake and polymer production rates (volumetric productivity). Without benefit of the stimulation effect (case of *HRT_m_* = 250 min in Figure 9), higher volumetric productivity results in a direct trade-off with loss of substrate utilisation efficiency. Increasing amounts of substrate are discharged in the effluent. With all other things begin equal, substrate losses are significantly mitigated if the biomass has been stimulated to higher respiration rate (case of *HRT_m_* = 5 min in Figure 9). For the modelled process scenario, a targeted organic loading rate of greater than about $f_{L}$ equal to 0.9 was a break point. Progressive losses in substrate utilisation efficiency result without further gains in volumetric productivity beyond the break point.

The stochastic modelling of the observed MMC biomass response in stimulation of respiration and substrate uptake rates suggests potential to understand and develop optimal operational conditions for robust continuous feed methods at industrial scale. Characteristics for any biomass may be readily determined by the methods of the respiration experiments that were replicated for both the laboratory and pilot scale SBR enrichment biomass sources in the present investigation. This kind of characterisation of the biomass dynamic response is recommended towards innovation in the bioprocess that can address challenges for maximising productivity and for avoiding growth of flanking populations.

In the present work, the dynamic response characterisation for two biomass sources revealed a similar inhibition effect of specific acid dosing (Figure 6B,C). This suggests a benefit of dosing substrate to higher concentrations of biomass. If biomass retention is by gravity separation, then recirculation and stimulation could be achieved by influent mixing with the return thickened biomass suspended solids coming from the clarifier. Higher suspended solids concentration brings added buffer capacity to the stimulation mixing zone.

Recirculation to *V_s_* using as low a *HRT_m_* as possible helps to initially stimulate all the process biomass as quickly as possible. Feeding exclusively to the stimulation zone becomes less critical to maintain if all the process biomass has been “stimulated”. Once all the process biomass is stimulated the process control objective is to ensure a feeding rate to sustain sufficient but still relatively low substrate concentration levels (ca. 5 to 25 mgCOD/L, based on the estimated *K_s_* of about 2 mgCOD/L). Thus a challenge is to establish feedback control for optimum feeding rate without over or under feeding the process.

In the model simulations, the required influent flow rate was calculated. In practice, the feeding rate must be determined from the process monitoring. Recirculating to the stimulation zone with known flows, mixing ratios and characteristic time constants, can serve needs for monitoring and feed rate feedback control [31]. *V_s_* and *V_m_* zones can be monitored in parallel with sensor signals (dissolved oxygen, pH, ORP, IR-spectroscopy, and so forth) that are related directly or indirectly to substrate concentrations, biomass respiration levels, and/or PHA content. The *V_s_* zone provides a dynamic reference measurement because water quality parameters, including but not limited to, buffer capacity, and ionic composition will change in time. Changes will be due to the feedstock water quality and also the intense PHA accumulation biological activity in *V_m_*. So-called soft-sensing process control strategies [53] are anticipated to become necessary. The process modelling methods applied in the present work can be adapted to test principles of control strategies for the polymer production. Addressing the challenges of developing robust and relevant strategies for the MMC accumulation process monitoring and control will be essential for successful industrial scale process implementations.

## 4. Conclusions

There are three main outcomes as conclusions from this study and with novelty in contributions from the investigation efforts to characterize and model dynamics of substrate uptake rate for MMCs during PHA accumulation:

1. A property of hysteresis in the dynamic response of MMCs storing PHA could be demonstrated for two distinct enrichment cultures using dissolved oxygen and chemical oxygen demand mass balance experiments.
2. This hysteresis could be modelled with readily identifiable parameters using Monod equations describing the distinct upshift and downshift dynamics in substrate uptake rates as a function of substrate concentrations and as a function of time. It was found that the substrate concentration, required to stimulate a substrate uptake rate, was higher than the substrate concentration required to maintain an attained substrate uptake rate.
3. The system of equations in numerical simulations suggest for an opportunity to exploit this property of hysteresis in industrial scale bioprocesses for PHA production. MMC PHA production processes can be operated with continuous feeding strategies, even with low concentration feedstocks. The model simulations found that engineered stimulation zones can be applied in continuous flow PHA production bioprocesses as a strategy to reach maximum possible performance in volumetric productivity without sacrificing performance in substrate utilization efficiency.

Applying ideas and principles revealed by the model simulations is a future challenge with applications in practice. Methods for process monitoring are required in combination with feedback control to maintain optimal organic loading rates during the PHA accumulation process.

## Figures and Tables

**Table 1:** Characterization of laboratory and pilot scale SBR MMC enrichment biomass with data from respiration experiments with oxygen and chemical oxygen demand mass balance modelling when active constant aeration was applied. Values in the brackets give the number of measurements or experiments used in estimation of means with standard deviations.

Parameter	Units	Laboratory Scale	Pilot Scale
Temperature	°C	24.6 ± 0.3 (24)	23.8 ± 0.6 (17)
pH (*t* = 0)	-	9.2 ± 0.1 (24)	9.0 ± 0.1 (17)
*X_a_*	gVSS/L	1.0 ± 0.1 (11)	1.71 ± 0.04 (2)
*k_a_*	1/min	1.11 ± 0.14 (24)	0.60 ± 0.15 (17)
*q_oe_*	mgO_2_/gVSS/min	0.64 ± 0.12 (24)	0.25 ± 0.07 (17)
*Y_os_*	gO_2_/gCOD	0.26 ± 0.02 (24)	0.23 ± 0.05 (17)
*k_s_*	mgCOD/L	2.0 ± 0.7 (24)	1.8 ± 0.4 (17)
*k_u_*	mgCOD/L	38 ± 7 (24)	20 ± 2 (17)
$q_{s}^{r}$	mgCOD/gVSS/min	7.4 ± 0.1 (24)	2.5 ± 0.2 (17)
$q_{s}^{u}$	mgCOD/gVSS/min	21.1 ± 1.1 (24)	6.8 ± 0.2 (17)

## Data Availability

The data presented in this study are available on request from the corresponding author.

## Abbreviations

The following abbreviations and symbols are used in this manuscript:

ATR-FTIR	attenuated total reflection-FTIR
BOD	biochemical oxygen demand
COD	chemical oxygen demand
DO	dissolved oxygen
FTIR	Fourier transform infrared spectroscopy
HRT	hydraulic retention time
MMC	mixed microbial culture
PHA	polyhydroxyalkanoate
PHB	polyhydroxybutyrate
SBR	sequencing batch reactor
SRT	solids retention time
VFA	volatile fatty acid
VSS	volatile suspended solids
WWTP	wastewater treatment plant
α	PHA accumulation inhibition exponent
$C_{o}$	DO concentration (mgO_2_/L)
$C_{o}^{*}$	apparent maximum DO (saturation) concentration (mgO_2_/L)
$C_{s}$	exogenous dissolved substrate concentration (mgCOD/L)
$C_{si}$	initial or peak upshift $C_{s}$ (mgCOD/L)
$1-f_{i}$	$q_{s}^{i}$/$q_{s}^{m}$
$k_{a}$	aeration oxygen mass transfer coefficient (1/min)
$k_{h}$	Haldane substrate inhibition constant (mgCOD/L)
$k_{i}$	substrate induction constant (mgCOD/L)
$k_{o}$	Monod apparent affinity constant on DO concentration (mgO_2_/L)
$k_{s}$	Monod apparent downshift affinity constant on substrate concentration (mgCOD/L)
$k_{u}$	Monod apparent upshift affinity constant on peak substrate concentration (mgCOD/L)
$Q_{oa}$	DO supply rate due to aeration (mgO_2_/L/min)
$Q_{oe}$	DO consumption rate due to endogenous respiration (mgO_2_/L/min)
$Q_{op}$	DO consumption rate due to stored PHA (mgO_2_/L/min)
$Q_{os}$	DO consumption reate due to substrate consumption (mgO_2_/L/min)
$q_{op}$	specific $Q_{o}p$ (mgO_2_/gVSS/min)
$q_{os}$	specific $Q_{o}s$ (mgO_2_/gVSS/min)
$q_{s}^{r}$	biomass resting specific substrate uptake rate (mgCOD/gVSS/min)
$q_{s}$	specific substrate uptake rate (mgCOD/gVSS/min)
$q_{s}^{e}$	maximum extant specific substrate uptake rate (mgCOD/gVSS/min)
$q_{s}^{i}$	initial maximum extant specific substrate uptake rate (mgCOD/gVSS/min)
$q_{s}^{m}$	maximum specific substrate uptake rate (mgCOD/gVSS/min)
*t*	time (minutes)
$q_{s}^{r}$	biomass maximum upshift in specific substrate uptake rate (mgCOD/gVSS/min)
$X_{a}$	active biomass concentration (gVSS/L)
$X_{p}$	PHA concentration (gCOD/L)
$Y_{os}$	yield of oxygen consumed with substrate (mgO_2_/mgCOD)
$C_{sf}$	influent feedstock substrate concentration (mgCOD/L)
$C_{sm}$	maintenance volume substrate concentration (mgCOD/L)
$C_{ss}$	stimulation volume substrate concentration (mgCOD/L)
$f_{L}$	applied fraction of maximum possible organic loading rate
$f_{PHAi}$	*i*th-active biomass element specific PHA level ($x_{p}$/$x_{a}$)
$f_{PHA}^{m}$	active biomass maximum PHA level for $x_{p}$/$x_{a}$
$HRT_{m}$	HRT in $V_{m}$ due to $Q_{m}$ (min)
$HRT_{s}$	HRT in $V_{s}$ due to $Q_{m}$ (min)
$n_{m}$	number of biomass elements in $V_{m}$
$Q_{f}$	substrate feed (and effluent) flow rate (L/min)
$Q_{m}$	recirculation flow rate (L/min)
$q_{si}^{m}$	*i*th-biomass element maximum specific substrate uptake rate (mgCOD/gVSS/min)
$V_{m}$	maintenance volume (L)
$V_{s}$	stimulation volume (L)
$x_{a}$	element specific active biomass (gVSS/element)
$x_{p}$	element specific PHA content (gPHA/element)
